# Parasitic Myoma After Morcellation

**DOI:** 10.4103/0974-1216.71612

**Published:** 2009

**Authors:** Rakesh Sinha, Meenakshi Sundaram, Smita Lakhotia, Pratima Kadam, Gayatri Rao, Chaitali Mahajan

**Affiliations:** Bombay Endoscopy Academy and Centre for Minimally Invasive Surgery (Beams Hospital), Mumbai, India

**Keywords:** Laparoscopic myomectomy, parasitic fibroid, retained fragment after morcellation

## Abstract

We report an interesting case of parasitic fibroid which developed from a morcellation remnant following laparoscopic myomectomy. The patient presented with incidental finding of pelvic mass in 2005. She underwent laparoscopic myomectomy for a myoma extending from the Pouch of Douglas to both sides of broad ligament. She subsequently presented with abdominal pain 3 years later in 2008. She underwent total laparoscopic hysterectomy with removal of broad ligament fibroids. During her hysterectomy, a right lumbar mass attached to the omentum was detected, which was excised laparoscopically. Histopathology of the mass confirmed it to be consistent with leiomyoma. This mass could probably be a morcellation remnant that has grown to this size taking blood supply from the omentum. We report this case to emphasize that all tissue pieces that are morcellated should be diligently removed. Even small bits displaced into the upper abdomen can result in parasitic fibroids. Thus, it can be concluded that parasitic myomas can arise from morcellated remnants and grow depending on the blood supply.

## INTRODUCTION

Parasitic myomas are rare but have been reported in literature. These could be myomas detached from the uterus, which have taken blood supply from adjacent organs or could be retained myoma fragments. Laparoscopic retrieval of specimen by morcellation aids in the removal of large specimens but incurs the risk of incomplete removal. These retained fragments can get dislodged in the peritoneal cavity and take blood supply from adjacent structures and grow. Some of them can cause symptoms, grow to any size and present as mass anywhere in the peritoneal cavity.

## CASE REPORT

A 42-year-old multiparous woman presented to us with complaints of pain in the abdomen of 6 months duration. She underwent laparoscopy for a pelvic mass 3 years before her present complaints. There was a 10 cm × 9 cm mass from the Pouch of Douglas extending into both broad ligaments. Laparoscopic myomectomy was done. The fibroid was degenerated. The specimen was retrieved by morcellation. The specimen weighed 620 g. She was asymptomatic for 3 years and presented to us with abdominal pain and fibroids detected on ultrasonography.

Clinical examination revealed a bulky uterus with a mass in the pouch of Douglas. Ultrasound revealed a mass of 6 × 5cm in the Pouch of Douglas and a 7 × 6 cm mass in the right lumbar region near the right kidney but separate from it. She did not have any complaints pertaining to the right lumbar mass.

**Figure 1:**
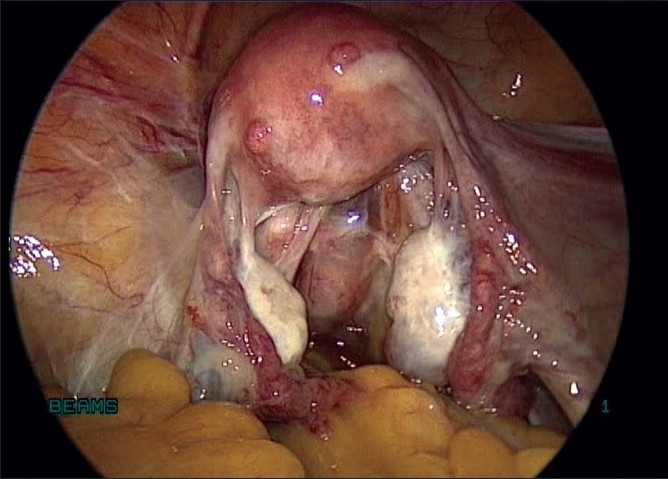
Bulky uterus with adnexa

**Figure 2:**
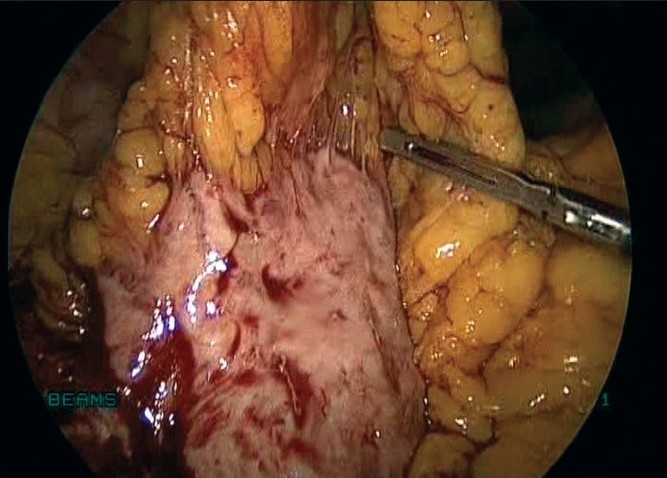
Parasitic myoma in the omentum

Laparoscopy was done with four ports, one primary supraumbilical and three accessory ports, two on the left and one on right side. Laparoscopy revealed a bulky uterus with small fibroids [Figure 1]. There was a 6 × 5 cm degenerated pelvic mass extending bilaterally in the broad ligament. On surveying the upper abdomen, she had a 7 × 6 cm mass engulfed completely by the omentum [Figure 2].

**Figure 3:**
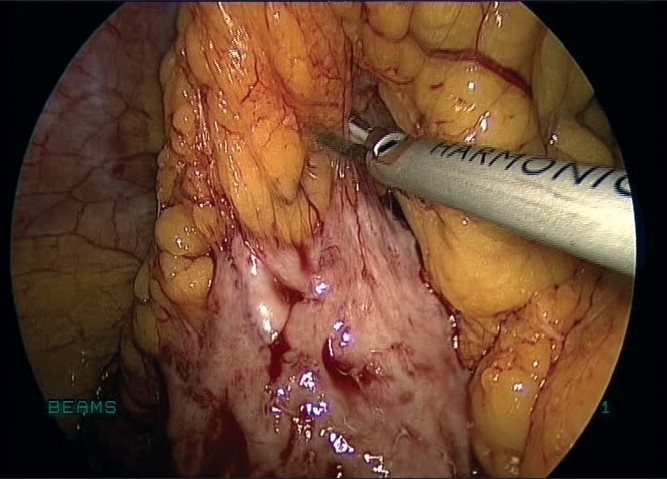
Myoma released with harmonic ultracision

**Figure 4:**
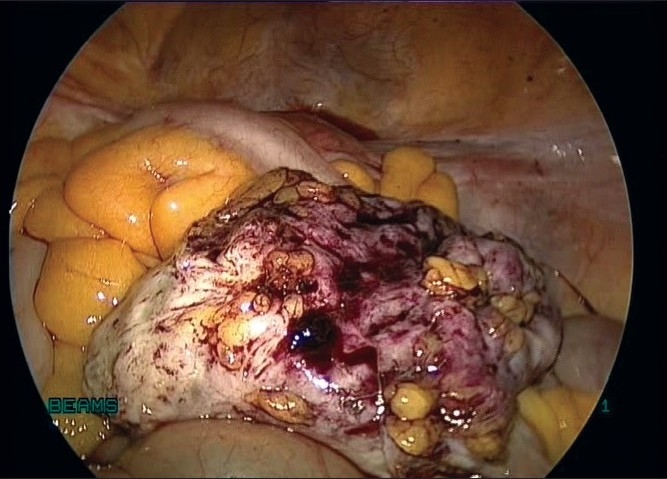
Myoma after complete excision

We proceeded with total laparoscopic hysterectomy with excision of the pelvic mass. The lumbar mass was dissected carefully from the omentum with the harmonic ultracision [Figure 3] and ensuring that it did not arise from the bowel or from any other structure. The uterus, pelvic mass and the right lumbar mass [Figure 4] were retrieved vaginally. The vaginal vault was closed with interrupted No.1 delayed absorbable sutures. The uterus and the pelvic mass weighed 300 g. The lumbar mass weighed 80g.

The specimen was sent for histopathology. The pelvic mass and the right lumbar mass were reported as leiomyoma. The patient has an unremarkable postoperative period and is asymptomatic till date.

## DISCUSSION

Leiomyomas are benign smooth muscle tumors clinically apparent in 20–25% of women of reproductive age. Advances in retrieval systems have made laparoscopic myomectomy a feasible option, irrespective of size, site or number of myomas.[1] After laparoscopy, the enucleated myoma can be retrieved by minilaparotomy, colpotomy or by morcellation into smaller fragments.

Tissue morcellation, especially in large myomas, may be very time consuming, and tissue pieces may be spread in the abdominal cavity. The advent of electromechanical morcellators have aided in the retrieval of large masses laparoscopically. It is essential for the surgeon to keep watching for falling pieces during morcellation and effort should be made to remove every single piece to prevent retained fragments.

These retained fragments usually get infarcted and present with abdominal pain, which necessitates immediate removal of the mass. There have been reports of retained tissue becoming necrotic and causing severe peritonitis.[2] Very rarely like in our case, the retained fragment can take blood supply from an adjacent organ and grow. They can cause symptoms or sometimes they can just be incidental finding on imaging. Asymptomatic fragments found as incidental findings could be mistaken for more ominous pathology such as pelvic or abdominal malignancies.

There is a report of a uterine leiomyoma particle growing in an abdominal wall incision after laparoscopic retrieval, suggesting inadvertent implantation at the site of removal through the trocar sleeve.[3] Hutchins and Reinoehl reported about a retained myoma after laparoscopic supracervical hysterectomy with morcellation. They retrieved a 5 × 4-cm infarcted myoma by exploratory laparotomy. The specimen was lost during morcellation.[4]

There are reports of myomas that have spontaneously lost their connections to the uterus and parasitized other blood supplies. The author has reported a case of parasitic myoma under the dome of the diaphragm.[5]

The exposure of these tumors to steroid hormones and growth factors plays an important role in their growth. The possibility of this lumbar mass in our case to be disseminated leiomyomatosis is ruled out as it was a single mass. It is most probably a retained morcellation fragment from previous myomectomy, which has become parasitic. The authors have also reported two cases of large multiple leiomyomas developing after laparoscopic hysterectomy.[6]

Given the potential sequelae of retained fragments, careful attention to remove all residual specimens is warranted. Placing the patient in reverse Trendlenburg position after morcellation and copiously irrigating the abdomen and pelvis may be helpful in washing small pieces into the pelvis.[7] After intraperitoneal lavage, a thorough evaluation of the abdomen and pelvis should be performed and any remaining tissue removed.

This case is reported to ensure that identification and removal of all fragments, however minute they are, is mandatory after morcellation. With the increase in laparoscopic debulking procedures for uterine surgery, the incidence in retained morcellated fragments of myomatous uterus will probably increase. Knowledge of this potential complication and the associated imaging findings, as well as the clinical history, is essential for the proper diagnosis of these lesions, whether or not the patients are symptomatic.

## References

[1] Sinha R, Hegde A, Mahajan C, Dubey N, Sundaram M (2008). Laparoscopic myomectomy: Do size, number, and location of the myomas form limiting factors for laparoscopic myomectomy?. J Minim Invasive Gynecol.

[2] Lieng M, Istre O, Busund B, Qvigstad E (2006). Severe complications caused by retained tissue in laparoscopic supracervical hysterectomy. J Minim Invasive Gynecol.

[3] Ostrzenski A (1997). Uterine leiomyoma particle growing in an abdominal wall incision after laparoscopic retrieval. Obstet Gynecol.

[4] Hutchins FL, Reinoehl EM (1998). Retained myoma after laparoscopic Supracervical hysterectomy with morcellation. J Am Assoc Gynecol Laparosc.

[5] Sinha R, Hegde A, Mahajan C (2007). Parasitic myoma under the diaphragm. J Minim Invasive Gynecol.

[6] Sinha R, Sundaram M, Mahajan C, Sambhus A (2007). Multiple leiomyomas after laparoscopic hysterectomy: Report of two cases. J Minim Invasive Gynecol.

[7] LaCoursiere DY, Kennedy J, Hoffman CP (2005). Retained fragments after total laparoscopic hysterectomy. J Minim Invasive Gynecol.

